# Heterostructural Mixed Oxides Prepared via ZnAlLa LDH or ex-ZnAl LDH Precursors—Effect of La Content and Its Incorporation Route

**DOI:** 10.3390/ma14082082

**Published:** 2021-04-20

**Authors:** Katarzyna Antoniak-Jurak, Paweł Kowalik, Wiesław Próchniak, Robert Bicki, Grzegorz Słowik

**Affiliations:** 1 Catalysts Department, Łukasiewicz Research Network-New Chemical Syntheses Institute, Al. Tysiąclecia Państwa Polskiego 13A, 24-110 Puławy, Poland; pawel.kowalik@ins.lukasiewicz.gov.pl (P.K.); wieslaw.prochniak@ins.lukasiewicz.gov.pl (W.P.); robert.bicki@ins.lukasiewicz.gov.pl (R.B.); 2 Department of Chemical Technology, Institute of Chemical Sciences, Faculty of Chemistry, Maria Curie-Sklodowska University in Lublin, Maria Curie-Sklodowska Sq. 3, 20-031 Lublin, Poland; grzegorz.slowik@poczta.umcs.lublin.pl

**Keywords:** non-stoichiometric zinc aluminate spinels, mixed metal oxides, heterostructural nanocomposites, surface reconstruction of ZnAl_2_O_4_

## Abstract

The effect of La content and its incorporation route on physicochemical properties of ZnO/Zn(Al,La)_2_O_4_ or La_2_O_3_–ZnO/ZnAl_2_O_4_ mixed oxides with a spinel structure obtained from ZnAlLa Layered double hydroxides (LDHs) or ex-ZnAl LDH materials was investigated. The heterostructural nanocomposites with the similar Zn/Al molar ratio and varied La content were prepared by two techniques: via co-precipitation and thermal treatment of ZnAlLa LDHs at 500 °C or via incipient wetness impregnation of ex-ZnAl LDHs with aqueous solutions of lanthanum nitrate and subsequent thermal treatment. The obtained series of materials were characterized by the following techniques: X-ray fluorescence (XRF), N_2_ adsorption (BET), X-ray diffraction (XRD), Fourier-transform infrared spectroscopy (FT-IR), thermogravimetric analysis with evolved gas analysis (TG/DTG/EGA), scanning transmission electron microscopy (STEM) energy-dispersive X-ray spectroscopy (EDS), high-resolution transmission electron microscopy (HRTEM) and Fourier-transform infrared spectroscopy (FFT). The evaluation of activity toward the high-temperature water gas shift (HT-WGS) within the temperature range of 350–420 °C was carried out on the basis of rate constant measurements in the kinetic mode using a differential reactor. The co-precipitation technique allowed for a better distribution of La in bulk and on the spinel surface than in case of lanthanum incorporation via impregnation. ZnO/Zn(Al,La)_2_O_4_ or La_2_O_3_–ZnO/ZnAl_2_O_4_ mixed oxides were characterized by moderate activity in the HT-WGS reaction. The results reveal that introduction of lanthanum oxide over 2.4–2.8 wt% induces the phase separation of the ZnAl_2_O_4_ spinel, forming ZnO on the ZnAl_2_O_4_ spinel surface.

## 1. Introduction

Layered double hydroxides (LDHs) are commonly applied as catalyst supports, catalyst precursors and nanocatalysts [1,2,3]. The uniqueness of LDHs is associated with an easy anionic metal complexes incorporation into the interlayer galleries of regular arrangement. An additional advantage of LDHs is the ability to incorporate a wide range of various cations with a similar ionic radius, e.g., Mg^2+^, Cu^2+^, Ni^2+^, into the brucite-like layers [4]. During thermal treatment, they decompose forming nano-sized simple and mixed oxides with unique properties. The characteristic features of resulting materials are high specific surface area, homogeneous distribution of components (even on the atomic level) and the so called memory effect involving the recovery of the primary structure in contact with an aqueous salt solution [5].

Mixed Zn–Al oxides obtained from LDHs are used in a number of applications as sensors, high temperature ceramic material, photocatalysts [6], supports and catalysts for various chemical reactions [2]. In the preparation process, numerous synthesis methods are applied, i.e., coprecipitation [7,8], hydrothermal [9], microemulsion techniques [10], allowing to obtain the specific defective structures with enhanced catalytic properties [11]. Stability of ex-LDH catalytic systems can be increased by modification with rare earth metals (La, Ce) of low electronegativity [12]. Their presence reduces mobility of surface atoms and thus it stabilizes the catalyst structure. Lanthanum oxide is one of the most effective stabilizers of structural properties of metallic and oxide catalysts containing alumina, as it inhibits the phase transition of γ-Al_2_O_3_ to α-Al_2_O_3_ [13]. According to Liu [14], it can be concluded that La is easily incorporated into the spinel-like structure of Mg_x_Al_y_O_z_. The consequence of this is the increase of an average pore size and durability in a steam-rich atmosphere. Similar effects of La doping may be expected in the case of spinel Zn_x_Al_y_O_z_.

In the preparation of ZnAlLa materials, numerous synthesis methods are applied. Xie et al. [15] studied systems with the same molar ratio (M^2+/^M^3+^ = 2) and variable La content (La^3+^/Al^3+^ molar ratio in the range from 0 to ∞) obtained using precipitation with sodium hydroxide (at low CO_3_^2−^ concentration in the reaction environment) at variable pH (ranging from 2 to 13) and then subjected to hydrothermal processing (110–140 °C) and calcination (100–700 °C). The other variant of Zn–Al–La synthesis was proposed by Liu [14]. That catalyst preparation route (molar ratio of Zn/Al = 0.5 and La content in the range of 1.5–30 wt%) involved coprecipitation of the hydroxycarbonate precursor at pH ranging from 8.0 to 9.0, removal of Na^+^ and subsequent precursor calcination at 700 °C.

The catalytic effect of lanthanum-doped Zn–Al systems was also studied in esterification. According to Tzompantzi et al. [16,17] La incorporation at the stage of synthesis of Zn–Al–La LDHs via the urea route increases activity of the Zn–Al oxide catalyst towards the esterification reaction. The object of the study was systems with a constant Zn/Al = 2 molar ratio, in which the La share was up to 3 wt%. Oxide materials obtained by ZnAlLa LDH calcination at 400 °C displayed a significantly smaller surface area but increased pore volume with an increase in the La_2_O_3_ content. A feature that particularly influences the better catalytic properties of Zn–Al–La systems in relation to the unmodified material is higher surface alkalinity, especially the presence of strongly alkaline surface centers. The authors of the paper explained the creation of such centers with the intensive interaction that occurred on the ZnO–La_2_O_3_ interface. Their presence determines better catalytic properties of Zn–Al–La systems in the esterification reaction of oleic acid fatty grass and phenol photomineralization in comparison with Zn–Al systems.

Another approach to synthesis of the ZnAlLa nanospinel was presented by Shariatinia et al. [18] proposing the use of gel combustion with poly(vinyl alcohol) or glycerin to obtain Zn–Al–La expressed in the following molar ratios: Zn/Al = 2.2, La/Al = 2.5.

Attempts to obtain effective catalysts for methanol steam reforming based on Zn–Al–La systems and with the same composition and obtained analogously using gel combustion and then supported on cordierite structural reactors were carried out by Khani et al. [19,20]. Such systems display favorable thermal stability which is proved with a relatively low decrease of the specific surface area at high temperatures (1000 °C). Based on the Zn–Al–La spinel, the copper catalyst was prepared which, after placing it on a monolith, appeared to be highly effective in methanol steam reforming.

To the best of our knowledge, the catalytic properties of Zn–Al–La materials, especially the effect of lanthanum on the efficiency and durability at high-temperature water–gas shift (HT-WGS) conditions, have not been studied so far.

The water–gas shift reaction is an exothermic reaction, and CO conversion is favored at lower temperatures. The process is carried out to enrich the synthesis gas in hydrogen with the simultaneous removal of carbon dioxide according to the following reaction: CO + H_2_O ⇄ CO_2_ + H_2_ (∆H (298K) = −41.1 kJ/mol). Usually, the WGS reaction is conducted in two adiabatic reactors arranged in series, which are the high-temperature WGS (HT-WGS, 320–450 °C) and the low-temperature WGS (LT-WGS, 200–250 °C), to maximize the CO conversion. In our previous publication [21], it was shown that ecofriendly K-decorated mixed oxides (Zn–Al) with a spinel-type structure obtained via co-precipitation and thermal treatment of ZnAl LDHs (with compositions of (Zn/Al)_mol_ = 0.3, 0.5 and 1.0) catalyze the HT-WGS reaction effectively. The highest activity was found for the Zn/Al molar ratio of 0.5 corresponding to spinel stoichiometry. In spite of their high activity, potassium-promoted Zn–Al systems display different thermal stability. It seems that this fact can be related to alkali mobility and tendency to surface migration. The use of the heterostructural nanocomposite of ZnO/Zn(Al,La)_2_O_4_ or La_2_O_3_–ZnO/ ZnAl_2_O_4_ allows for better stabilization of the alkali that are used as promotors of HT-WGS catalysts.

The presented work describes the effect of La content and its incorporation route on the physicochemical properties of ZnO/Zn(Al,La)_2_O_4_ or La_2_O_3_–ZnO/ZnAl_2_O_4_ mixed oxides with the spinel structure. Ternary Zn–Al–La oxide systems were prepared by coprecipitation and thermal treatment of ZnAlLa LDHs at 500 °C and via impregnation of ex-ZnAl LDHs with the lanthanum nitrate aqueous solution and subsequent thermal treatment at 500 °C. The obtained precursors and their calcination products were analyzed in terms of their physiochemical properties using X-ray fluorescence (XRF), N_2_ adsorption (BET), X-ray diffraction (XRD), thermogravimetric analysis with evolved gas analysis (TG/DTG/EGA), scanning transmission electron microscopy(STEM)– energy-dispersive X-ray spectroscopy (EDS), Fourier-transform infrared spectroscopy (FT-IR), high-resolution transmission electron microscopy (HRTEM) and fast Fourier transform (FFT). The activity of ZnAl_W_k__xLa and ZnAl_I_k__xLa series was evaluated in the HT-WGS reaction.

## 2. Materials and Methods

### 2.1. Preparation of La-Doped Zn–Al Materials

Two series of La-modified Zn–Al materials were prepared.

Series I was prepared via co-precipitation and thermal treatment of ZnAlLa LDHs at 500 °C. Zn–Al–La LDH materials with a similar molar ratio of Zn/Al (~0.7) and with different nominal content of La (from 1 to 9 wt%) were prepared by coprecipitation method from aqueous solutions of zinc, aluminum and lanthanum nitrate with the aqueous solution of Na_2_CO_3_ + NaOH at 75–80 °C and pH in the range of 7.5–8. After complete precipitation, the slurry was aged at 80 °C for 0.5 h under vigorous stirring. The solids obtained were thoroughly filtered and washed and then dried at 100 °C and subsequently calcined at 500 °C for 4 h. After drying, the prepared samples were marked with the following symbols: ZnAl_W_1La, ZnAl_W_2La, ZnAl_W_3La, ZnAl_W_4La, ZnAl_W_5La, and the corresponding calcination products were marked with the following symbols: ZnAl_W_k__1La, ZnAl_W_k__2La, ZnAl_W_k__3La, ZnAl_W_k__4La, ZnAl_W_k__5La.

Series II was prepared via impregnation with aqueous solutions of La(NO_3_)_3_ of ex- ZnAl LDH materials.

Preparation of the ZnAl LDH precursor with the composition expressed as a molar ratio of Zn/Al (0.65–0.7) was carried out by co-precipitation of ZnAl LDHs through simultaneous dosing of two streams, the (Zn(NO_3)2_, Al(NO_3_)_3_) aqueous solution and the Na_2_CO_3_ + NaOH aqueous solution, to the precipitation reactor. Synthesis was carried out at the temperature of 75–80 °C and pH in the range of 7.5–8. The obtained residues were thoroughly washed and filtrated and then were dried at 105 °C and calcined at 500 °C for 4 h. In the subsequent stage, the materials were subjected to impregnation with aqueous solutions of lanthanum nitrate with concentrations so that the nominal La content in the final materials ranging from 1 to 9 wt% could be achieved. After rehydration, the samples were dried and calcined at 500 °C for 4 h. After drying, the prepared samples were denoted with the following symbols: ZnAl_I_1La, ZnAl_I_2La, ZnAl_I_3La, ZnAl_I_4La, ZnAl_I_5La, and the corresponding calcination products were denoted with the following symbols: ZnAl_I_k__1La, ZnAl_I_k__2La, ZnAl_I_k__3La, ZnAl_I_k__4La, ZnAl_I_k__5La.

### 2.2. Material Characterization

The chemical composition of the samples was determined using the WD XRF method with an Axios mAx spectrometer (Malvern Panalytical, Malvern, UK) equipped with a 4 kW Rh SST-mAx lamp (Malvern Panalytical, Malvern, UK).

The XRD measurements were performed on an Empyrean (Malvern Panalytical, Malvern, UK) system (Bragg–Brentano geometry) equipped with a PIXcel3D detector ((Malvern Panalytical, Malvern, UK), using Cu Kα radiation (λ = 1.542 Å) and operating at 40 kV and 40 mA.

Fourier-transform infrared spectroscopy (FT-IR) measurements were carried out using Nicolet 8700A (Thermo Scientific, Waltham, MA, USA) equipped with a diamond crystal (Smart Orbit TR diamond ATR, Thermo Fisher Scientific, Waltham, MA, USA). Infrared spectra were obtained in the 4000–400 cm^−1^ spectral range.

The thermal decomposition with evolved gas analysis (TG/DTG/EGA) studies were carried out using a STA 449 Jupiter thermal analyzer (Netzsch,, Frankfurt, Germany) coupled with a QMS 430C Aëolos mass spectrometer (Netzsch, Germany). The measurements were performed in a helium stream, increasing the temperature range up to 800 °C with a rate of 10 °C/min. The changes of the sample weight and m/z signals (m/z = 18, 28, 30, 46) were constantly monitored.

The specific surface area (S_BET_) and the total pore volume (TPV) were determined using an ASAP^®^ 2050 Xtended Pressure sorption analyzer (Micromeritics Instrument Co., Norcross, GA, USA) from N_2_ adsorption and desorption isotherms at the temperature of −196 °C for p/p0 ≤ 0.99 using the BET adsorption model and BJH (Barret–Joyner–Halenda) transformation for mesopore characteristics.

The elemental mapping (STEM–EDS) of materials was conducted using a Tecnai G2 20 X-TWIN transmission electron microscope (FEI, Hillsboro, OR, USA). The sample mapping (determination of element distribution in the sample) was carried out using STEM and recording EDS spectra from each spot corresponding to pixels of the map, point after point. The collected maps were presented as a pixel matrix with a color of the mapped element and intensity corresponding to the content of the particular element.

HRTEM images were collected using a 60–300 kV Titan G2 transmission electron microscope (FEI, Hillsboro, OR, USA) equipped with a FEG (field emission gun), a monochromator, the system of three optical condensers, objective lenses, an image corrector (Cs corrector), a HAADF detector and an EDS (energy-dispersive X-ray spectroscopy) EDAX spectrometer with a Si(Li) detector. Microscopy studies were carried out at accelerating voltage equaling to 300 kV. Phase separation (crystal lattice of ZnAl_2_O_4_ and crystal lattice of ZnO) were performed with the FFT using masking available with the Gatan Digital Micrograph software package (Gatan, Inc., Pleasanton, CA, USA).

Activity in the high-temperature water–gas shift reaction (HT-WGS) was measured in the kinetic regime using a differential 4-channel reactor (conversion α < 0.1) under the following conditions: catalyst grain size (mm): 0.16–0.25, sample mass: 0.4 g, pressure (MPa): 2.5, temperature (°C): 330–400, gas flow (Ndm^3^/h): 60, gas composition (vol.%): CO/CO_2_/H_2_ = 3.9/9.6/86.5, saturated with (H_2_O/gas)_mol_ = 1.1 (steam). The stabilization time for each measurement was 2 h; then, the analyses of effluent gases were carried out using a GC (Shimadzu, Canby, OR, USA) equipped with a FID and a carbon sieve mesh: 100/120, length: 2 m, ID: 2 mm. The Shimadzu GC was calibrated for reactants (except for the steam; it was separated/condensed) from the effluent stream and a dry gas sample was injected to the GC system using certified gas mixtures containing 4/20/10 vol.% of CO/CH_4_/CO_2_ from Air Liquide (Pulawy, Poland). The HT-WGS reaction rate constant was calculated using the equation: r_HT-WGS_ = k_0_∙e^−(∆E/RT)^ × p_CO_^a^ × p_H2O_^b^ × p_H2_^c^ × p_CO2_^d^ × (1 − β), where:

rHT-WGS—WGS reaction rate (Ndm^3^∙g_cat_^−1^∙h^−1^);

k_0_—preexponential factor;

∆E—apparent activation energy;

pi—partial pressure of component “i” (MPa);

a, b, c, d—reaction order of CO, H_2_O, H_2_, CO_2_, respectively;

k—WGS rate constant (Ndm^3^∙gcat^−1^∙h^−1^∙at^−x^);

β—approach to equilibrium, β is defined as pCO_2_ × pH_2_ × pH_2_O^−1^ × pCO^−1^ × Kp^−1^;

K_p_—the equilibrium constant for the WGS reaction.

The exponents for H_2_O, CO_2_ and H_2_ were in the range of 0.02 ± 0.06, −0.10 ± 0.02, −0.22 ± 0.04, respectively [22].

## 3. Results and Discussion

### 3.1. Physicochemical Parameters of ZnAl_W_xLa and ZnAl_I_xLa Materials

**Figure 1:**
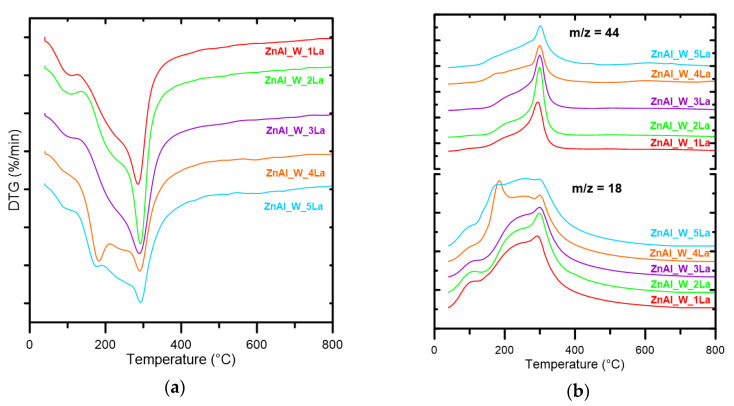
DTG (**a**) and EGA (**b**) curves for the series of ZnAl_W_xLa materials.

Table 1 shows the total weight loss in the temperature range up to 800 °C and the temperature corresponding to the maximum rate of decomposition (T_max_) of ZnAl_W_xLa and ZnAl_I_xLa materials. DTG/MS patterns for the series of ZnAl_W_xLa materials are presented in Figure 1. Two major stages of decomposition processes may be distinguished from DTG/EGA curves regardless of La concentration: low- (100–200 °C) and medium-temperature processes (210–410 °C). H_2_O and CO_2_ are the only decomposition products. Regardless of the lanthanum content, all the materials undergo complete thermal decomposition in the temperature range up to 410 °C. In the first stage, physically bound water is desorbed and simultaneous dehydration of interlayer spaces takes place, which is evidenced with an intensive m/z = 18 signal coming from water and a lack of distinct EGA m/z = 44 bands corresponding to CO_2_. In the second stage, in the range up to 410 °C, dehydroxylation of brucite-like layers and decarbonization with the release of CO_2_ take place. Less intense and sharp DTG bands are observed for the increased La content.

**Figure 2:**
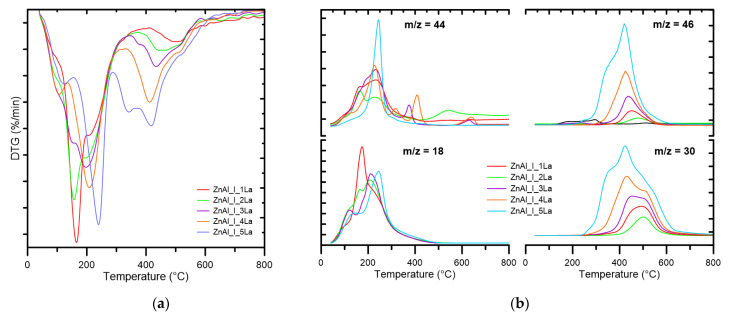
DTG (**a**) and EGA (**b**) curves for the series of ZnAl_I_xLa materials.

DTG/MS patterns of ZnAl_I_xLa series are presented in Figure 2a,b. Thermal decomposition of ZnAl_I_xLa series is more complex and proceeds at higher temperatures (100–680 °C). Three major stages can be distinguished: low- (100–200 °C), middle- (200–450 °C) and high-temperature (450–680 °C). Gaseous products of decomposition are H_2_O, CO_2_ and nitrogen oxide with the corresponding signals m/z = 30 (NO) and m/z = 46 (NO_2_). In the first stage, the release of H_2_O and simultaneous dehydration of interlayer spaces proceeds. In the next stage (200–450 °C), dehydration and decomposition of carbonates and nitrates intercalated between the layers of rehydrated ex-hydrotalcite-like mixed Zn–Al–La oxides take place.

The intensity increase of bands m/z = 30 and m/z = 46 is observed with the increase in lanthanum content and a significant increase and shift of maximum intensity towards lower temperatures. They correspond to nitrogen oxides being the products of thermal decomposition of lanthanum nitrate. The third stage involves further decomposition of NO_3_^−^ anions with the release of NO and NO_2_. The processes occurring over 600 °C have low intensity and are accompanied by CO_2_ release from decomposition of durable carbonates, the so called high-temperature carbonates (HT-CO_3_). In case of the ZnAl_I_xLa series, we observed the memory effect involving the recovery of the primary layer structure of the precursor as a result of the contact between the Zn–Al mixed oxide and the aqueous solution of lanthanum salt.

The amount of La added has an impact on the reconstruction process of meixnerite-like and hydrotalcite-like layer structures. Moreover, with the increase in La content, durability of these structures also increases, which is reflected in a slight but noticeable increase of decomposition temperatures for these materials (see Table 1).

### 3.2. Structural and Textural Properties of ZnAl_W_xLa and ZnAl_I_xLa Materials

**Figure 3:**
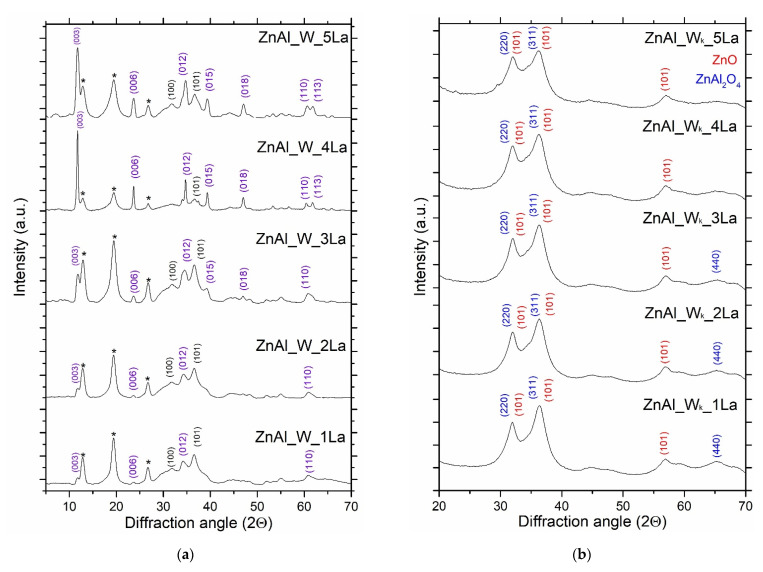
XRD patterns of ZnAl_W_xLa (**a**) and ZnAl_W_k__xLa (**b**) materials: Zn_0.6_Al_0.4_(OH)_0.2_·0.5H_2_O phase peaks at 2θ 11.7° (003), 23.6° (006), 34.7° (012), 39.1° (015), 46.6° (018), 60.5° (110) and 61.6° (113); 11.79°, 23.69°, 34.78°, 39.47° (015), 46.6° (018), 60.5° (110), 61.6° (113); (*) Zn_5_(CO_3_)_2_(OH)_6_ phase peaks at 2θ 12.8°, 19.2° and 26.5°; ZnO phase peaks at angles 2θ 31.8° (100), 36.1° (101).

The XRD patterns of ZnAl_W_xLa series are presented in Figure 3a. For the series of ZnAl_W_xLa materials, a well-structured phase of the hydrotalcite-like structure of the formula Zn_0.6_Al_0.4_(OH)_0.2_·0.5H_2_O is dominant, which is evidenced by characteristic peaks at 2θ 11.7°, 23.6°, 34.7°, 60.5°, corresponding to the diffraction on the planes (003), (012), (110), (113), respectively. Additionally, for materials with the highest content of La (ZnAl_W_4La, ZnAl_W_5La), planes (015), (018) and (113) are exposed at 2θ 39.1°, 46.6°, 61.6°. All the materials also contain complex zinc carbonate (Zn_5_(CO_3_)_2_·(OH)_6_) as evidenced by the characteristic peaks at 2θ 12.8°, 19.2° and 26.5° and trace amounts of ZnO. No phases corresponding to lanthanum compounds for the whole range of lanthanum content of the ZnAl_W_xLa series were found. It can be concluded that La was incorporated in the ZnAl LDH lattice.

The peaks characteristic of ZnAl LDH materials disappeared after calcination at 500 °C due to the collapse of lamellar structures (Figure 3b). In the phase composition of ZnAlLa_W_k__xLa materials, diffraction peaks at 2θ 31.2°, 36.7°, 65.0° and at 34.4°, 36.3° were observed, which are characteristic of nanocomposites of poorly crystallized mixed oxides (both ZnO and ZnAl_2_O_4_). No phases corresponding to lanthanum compounds in materials of the ZnAl_W_k__xLa series were found. Additionally, with the increase in the La content, peak broadening was observed, which indicates the decrease in the degree of nanocrystallinity of the mixed oxides.

As presented in Table S1, there was no influence of the lanthanum content on the size of ZnAl_2_O_4_ nanocrystallites. However, with the increase in the La content, a clear effect of decrease in the ZnO crystallite size was observed. The crystallinity decrease was probably caused by partial amorphization of ZnO. These observations are consistent with the data presented in the literature [15]. No phases corresponding to lanthanum oxides for the whole range of the lanthanum content of the ZnAl_W_k__xLa series were found. It can be concluded that La was incorporated in the bulk zinc alumiante spinel framework.

**Figure 4:**
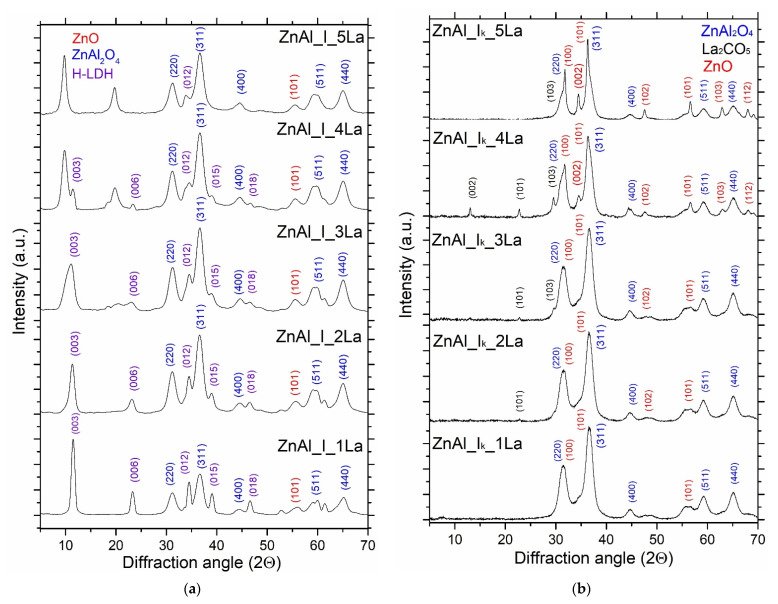
XRD pattern of the ZnAl_I_xLa (**a**) and ZnAl_I_k__xLa (**b**) materials.

For ex-hydrotalcite-like mixed Zn–Al oxides (ZnAl_I_xLa), due to impregnation with the aqueous lanthanum nitrate solution and drying at 105 °C (Figure 4a), the reconstruction of the layered structure was visible, which was demonstrated with the presence of peaks characteristic of the hydrotalcite analog at 2θ 11.7° (003), 23.6° (006), 34.3° (012), 39.3° (015), 47° (018). Moreover, for the material of the highest La content (ZnAl_I_5La), peaks at 2θ 9.9°, 19.9°, 34.3°, 37.7°, 43.4°, 61.1° were identified, which confirmed the presence of complex basic nitrates of Zn and Al. It showed that the applied conditions of thermal processing (500 °C) of ZnAl LDHs do not lead to irreversible changes of the formed heterostructural ZnO/ZnAl_2_O_4_ mixed oxides. ZnO/ZnAl_2_O_4_ nanocomposites recalcined at 500 °C exhibit the capacity to recover the primary layered structure of LDHs by rehydration with aqueous La(NO_3_)_3_ nitrate.

Quite different XRD patterns correspond to the ZnAl_I_k__xLa materials (Figure 4b). No phases originating from lanthanum compounds were identified for the materials of both the ZnAl_I_k__1La and ZnAl_I_k__2La series indicating that the La content in the studied materials is too low or that lanthanum has been partially incorporated into the Zn–Al spinel structure simultaneously with the rehydration process. However, for a higher La content (ZnAl_I_k__3La, ZnAl_I_k__4La), lanthanum is crystallized as La_2_CO_5_, which is demonstrated with the appearance of weak reflections for angles 2θ 13.1°, 22.9° and 29.5°. Relatively narrow peaks at 2θ 31.2°, 36.7°, 44.6°, 59.1°, 65.0° corresponding with diffraction planes of (220), (311), (400), (511), (440) were observed, which can be attributed to ZnAl_2_O_4_. It is worth noticing that for a higher content of La, the appearance of additional peaks at 2θ 36.3°, 68.0° corresponding to diffraction on planes (002), (103), (112) characteristic of the ZnO phase is visible.

As presented in Table S1, an evident effect of lanthanum content on ZnO nanocrystalite size (from 2.5 to 11.9 nm) was observed. The consequence of increase in the ZnO nanocrystalite size is a visible asymmetry of these peaks at 31.17°–33° and 35°–39°, indicating the presence of La_2_O_3_–ZnO/ZnAl_2_O_4_ mixed oxides. With the increase of the La content, there is an increased asymmetry between these peaks. However, splitting of heterojunction structures of mixed oxides La_2_O_3_–ZnO/ZnAl_2_O_4_ was not observed.

However, with the increase of the La content, no change in the size of ZnAl_2_O_4_ nanocrystallites was observed.

**Figure 5:**
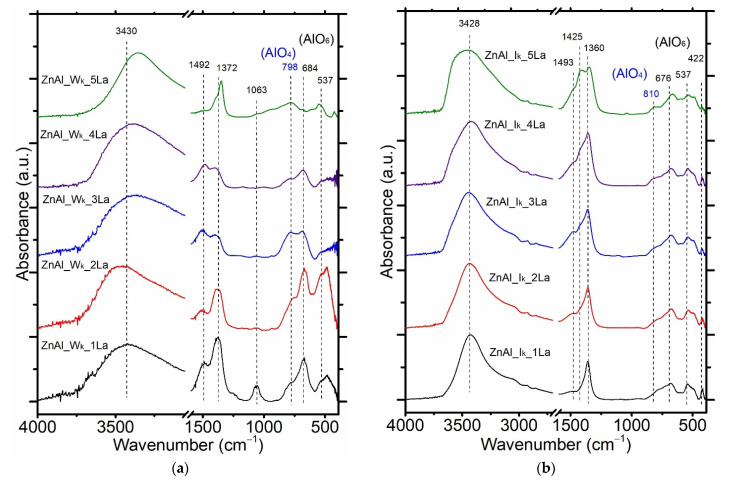
FT-IR spectra of the ZaAl_W_k__1La (**a**) and ZnAl_I_k__xLa (**b**) series of materials.

The structural properties of the ZnAl_W_k__xLa and ZnAl_I_k__xLa materials were also investigated by FT-IR (see Figure 5a,b). For the series of ZnAl_W_k__xLa and ZnAl_I_k__xLa materials, bands at a wavenumber range of 3700–2800 cm^−1^ were attributed to O–H bonds characteristic of surface OH groups.

For the series of ZnAl_W_k__xLa materials, the bands at 1372 and 1492 cm^−1^ characteristic of CO_3_^2−^ stretching vibrations were evidenced. In case of the ZnAl_I_k__xLa samples, the bands at wavenumbers of 1550–1300 cm^−1^ characteristic of antisymmetric stretching vibrations of CO_3_^2^^−^ intercalated between the layers of the ex-hydrotalcite-like mixed Zn–Al oxides were visible. The intensity of these bands changed with the increase of the La content. As presented in Figure 5a for the series of ZnAl_W_k__xLa materials, asymmetry of bands located in the range of 550–850 cm^−1^ indicates the presence of Al coordinated as AlO_6_ or AlO_4_. Bands at 684 cm^−1^ and 537 cm^−1^ corresponded to stretching vibrations of Al–O, whereas the band at 422 cm^−1^ could be attributed to bending O–Al–O bonds with octahedral coordination (AlO_6_). Bands at 798 cm^−1^ corresponded to Al–O bonds with tetrahedral coordination (AlO_4_). However, with the increase of the La content, the decrease of bands mentioned was evidenced, which suggests the structural evolution of ZnO–ZnAl_2_O_4_. For the series of ZnAl_I_k__xLa samples, the intensity of the bands located in the range of 550–850 cm^−1^ was relatively low.

Table 2 shows the total La content (wt%) for the series of ZnAl_W_k__xLa and ZnAl_I_k__xLa materials and the content of Zn and Al expressed with the Zn/Al molar ratio. The desired concentrations of the modifier and main components were verified by means of XRF measurements. For the series of ZnAl_W_k__xLa and ZnAl_I_k__xLa materials, a good agreement between the actual and nominal composition was achieved. Additionally, Table 2 presents textural parameters of ZnAl_W_k__xLa and ZnAl_I_k__xLa. The modification of the ZnAl_W_k__xLa series by introduction of a small amount of La via coprecipitation led to insignificant changes of textural properties. Both changes of the specific surface area and pore volume due to lanthanum compound addition (from 0.9 to 8.5 wt% La) were of inconclusive nature. The series of ZnAl_W_k__xLa materials with a relatively large specific surface area (in the range from 150 to 162 m^2^/g) and pore volume (in the range from 0.29 to 0.34 cm^3^/g) were obtained. For the series of ZnAl_I_k__xLa materials (La incorporated by impregnation), significantly smaller specific surface areas were obtained, including more than twice as small (for 8.7 wt% La–ZnAl_I_k__5La) in comparison with the materials prepared via the coprecipitation route. As the La content increased, the specific surface area decreased significantly, while the total pore volume increased. At relatively low concentrations of lanthanum (1.9–2.8 wt%), beneficial influence of La on the total pore volume increase was revealed. However, with further increase of the lanthanum content, the opposite effect was observed, i.e., the TPV decrease.

Tzompantzi et al. [16] studied the effect of Zn–Al modification by introduction of La at the stage of ZnAlLa LDH precursor synthesis via precipitation with the urea route and thermal treatment. Oxide materials obtained by calcination of the Zn–Al–La LDH precursor at 400 °C exhibited a significantly smaller surface area but an increased pore volume with an increase in the La_2_O_3_ content. Liu et al. [14] observed that for low content of La, the specific surface area appeared to be significantly smaller as compared to the reference ZnAl_2_O_4_, but further La content increase (at least 10 wt%) caused the reverse effect, that is, a significant specific surface area increase. The beneficial effect of La on the volume and average pore size was also observed. Authors relate this fact to the positive effect of La on the activity of modified ZnAl_2_O_4_ spinels in the trans-esterification reaction.

The pore size distributions for the series of ZnAl_W_k__xLa and ZnAl_I_k__xLa materials are presented in Figure S1. The pore systems of both the ZnAl_W_k__xLa and ZnAl_I_k__xLa series were complex and comprised three types of pores: mesopores with the diameter ranging from 2 to 10 nm, wide mesopores with a dominant diameter of 40–45 nm and macropores with a dominant diameter of 130–140 nm. The materials of ZnAl_Wk_xLa were characterized by a lower total pore volume as compared to the materials of ZnAl_I_k__xLa series and a similar pore distribution.

### 3.3. Suface Morphology of Mixed Oxides Obtained from ZnAlLa LDHs and ex-ZnAl LDHs

STEM–EDS images of selected materials of the ZnAl_W_k__xLa and ZnAl_I_k__xLa series are presented in Figures S2–S5. The STEM–EDS elemental maps show dispersion of Zn (yellow), Al (violet), O (red) in the investigated series of materials. Moreover, La (light blue) coverage and surface location are presented. STEM–EDS results for ZnAl_W_k__1La (Figure S2) indicate good dispersion of Zn and Al and a relatively uniform distribution of lanthanum over the surface of heterostructural mixed oxide nanoparticles. In case of higher concentrations of lanthanum (Figure S3), EDS maps revealed an apparently larger lanthanum surface coverage of nanoparticles of mixed oxides related to the intensity of the turquoise color on the EDS maps. Moreover, small agglomerates are locally visible.

XRD and DTG results showed that mixed oxides are capable of reconstructing the original LDH layer structure upon contact with an aqueous solution and a variety of anions can be intercalated into the recovered LDHs. Since the rehydration process, semi-homogenous distribution of La was evidenced in final materials. STEM–EDS studies of ZnAl_I_k__1La and ZnAl_I_k__5La materials showed significant differences in distribution. In case of ZnAl_I_k__1la, lanthanum nanoparticles were well dispersed. Relatively uniform distribution of lanthanum was present in the form of fine aggregates over the surface of mixed oxide nanoparticles. Lanthanum was distributed over ZnO nanoparticles and also decorated zinc aluminate spinel nanoparticles. For ZnAl_I_k__5La, the EDS maps showed non-uniform distribution and local agglomeration of lanthanum species.

The surface microstructure and morphology of the ZnAl_W_k__xLa and ZnAl_I_k__1La materials were investigated by HR-TEM (Figure S6 and S7). The series of ZnAl_W_k__xLa materials exhibited the needle morphology (see Figure S2). Some particles joined together to form elongated agglomerates.

**Figure 6:**
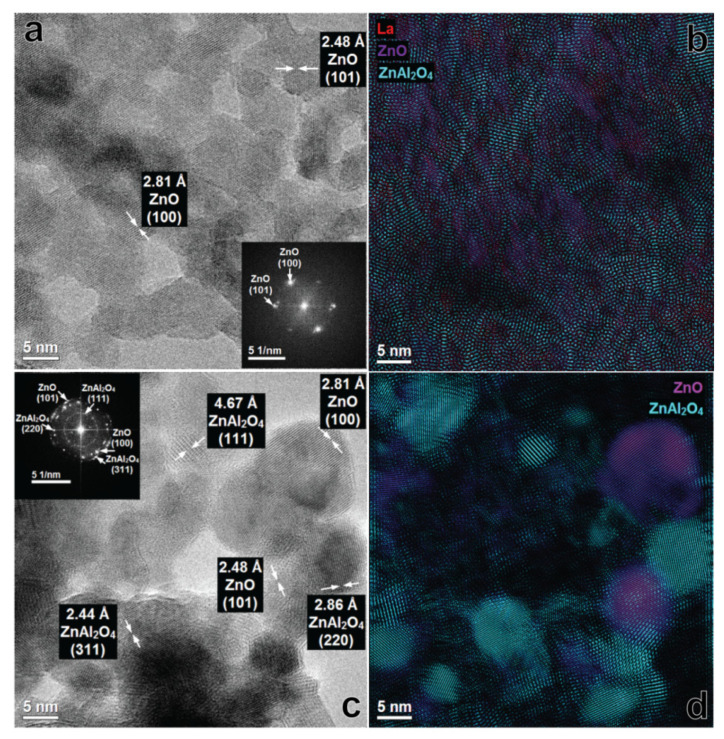
HRTEM image and FFT with phase identification of ZnAl_W_k__1La (**a**,**b**) and ZnAl_I_k__1La (**c**,**d**).

**Figure 7:**
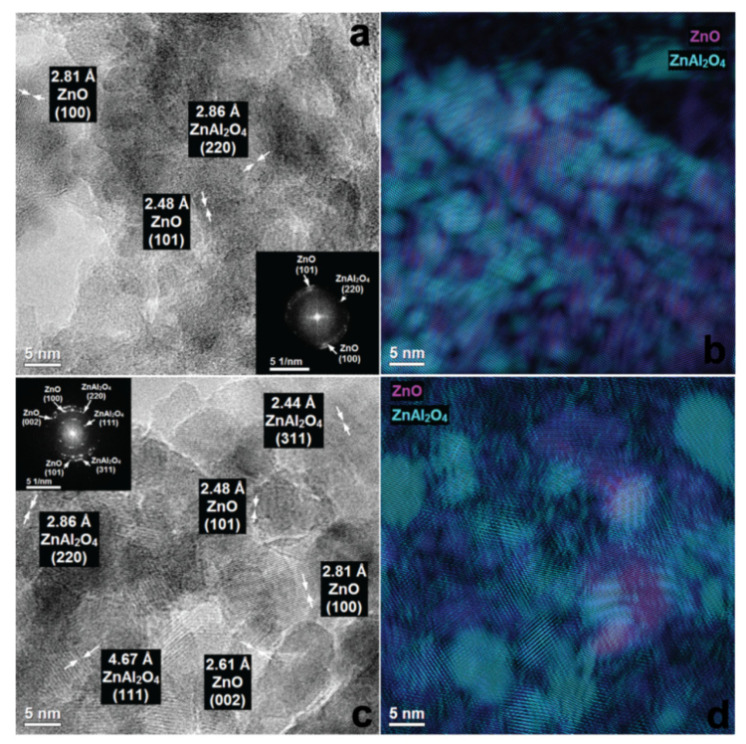
HRTEM images and FFT with phase identification of ZnAl_W_k__3La (**a**,**b**) and ZnAl_I_k__3La (**c**,**d**).

**Figure 8:**
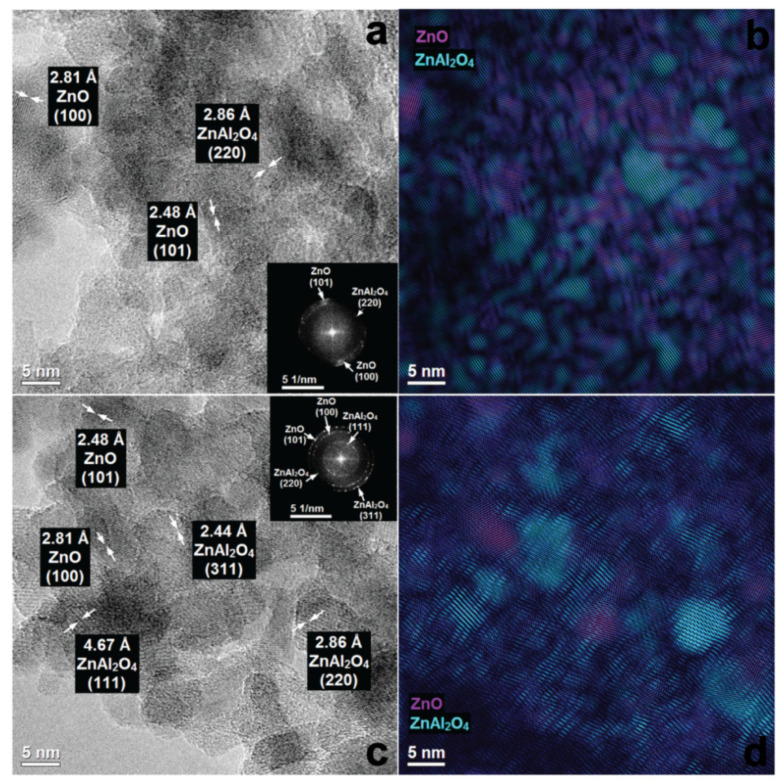
HRTEM images and FFT with phase identification of ZnAl_W_k__5La (**a**,**b**) and ZnAl_I_k__5La (**c**,**d**).

More detailed analysis of the ZnAl_W_k__1La materials by phase identification and separation revealed the particles of ZnO and ZnAl_2_O_4_ with a very small diameter (1–5 nm). Clear widening of the rings (Figure 6a) as well as broadening of XRD diffraction peaks (Figure 3a and Figure 6a) show low crystallinity of the material. With the increase of the lanthanum content, the surface morphology of the ZnAl_W_k__xLa series was changed. For ZnAlW_k__3La (Figure 7a,b), heterojunction structures of ZnO/ZnAl_2_O_4_ which are composed of ZnAl_2_O_4_ nanoparticles with an interplanar distance of 2.86 Å (220), 2.44 Å (311) and ZnO nanoparticles with an interplanar distance of 2.48 Å, 2.81 Å and lattice planes (100), (101) were visible. For ZnAl_W_k__5La, there were zinc-enriched areas in which very small ZnO nanoparticles were clearly segregated on the surface of zinc aluminate spinel nanoparticles. As presented in Figure 8a,b, the increase in the La content leads to the formation of amorphous ZnO on the ZnAl_2_O_4_ surface (see Figure 3b).

Phase identification and separation data analysis showed that no crystalline phases containing lanthanum were detected. It suggests that lanthanum is built into the zinc aluminate spinel framework.

The morphology of ZnAl_I_k__1La materials was substantially different from the ZnAl_W_k__xLa materials (see Figure S7 and Figure 6c,d). The ZnAl_I_k__1La material was composed of heterostructural nanocomposites with a more or less regular, round shape. One can distinguish nanoparticles of relatively large ZnO ((101) 2.48 Å, (100) 2.81 Å) and ZnAl_2_O_4_ ((111) 4.67 Å, (220) 2.86 Å, (311) 2.44 Å). With the increase of the lanthanum content (Figure 7c,d), the surface morphology of the ZnAl _I_k__xLa samples was changed. There were relatively large nanoparticles of ZnAl_2_O_4_ and ZnO, as well as zinc aluminate spinel nanoparticles which were partially covered with ZnO particles. As presented in Figure 8c,d for ZnAl_I_k__5La, distinct phase segregation of ZnO on ZnAl_2_O_4_ was observed. We speculate that the increase in the La content leads to the formation of segregated ZnO on the surface of ZnAl_2_O_4_.

### 3.4. Evaluation of ZnO/Zn(AlLa)_2_O_4_ and La–ZnO/ZnAl_2_O_4_ Mixed Oxides in the High-Temperature Water–Gas Shift Reaction

The catalytic activity of ZnO/Zn(AlLa)_2_O_4_ and La–ZnO/ZnAl_2_O_4_ mixed oxides in the HT-WGS was studied within the temperature range of 350−420 °C. Table 3 presents HT-WGS reaction rate constants for the ZnAl_W_k__xLa and ZnAl_I_k__xLa materials. The series of ZnAl_W_k__xLa and ZnAl_I_k__xLa mixed oxides presented a similar activity level in the HT-WGS reaction at 350 °C (at 350 °C, the reaction rate constant of series II is, on average, greater by ca. 30%). The differences of rate constant values for particular ZnAl_W_k__xLa and ZnAl_I_k__xLa materials were more visible at higher temperatures (370–420 °C).

For the content of La increased up to 1.9–2.8 wt%, the catalytic activity for the series of ZnAl_I_k__xLa materials significantly increased. For ZnAl_I_k__2La and ZnAl_I_k__3La, reaction rate constants at 420 °C have the value of approx. 14.1 and 13.8 (Ndm^3^∙g_cat_^−1^∙h^−1^∙at^−0.95^), respectively. The increase of the lanthanum content (above 2.8 wt%) leads to adverse effects, i.e., to the decrease of reaction rate constants.

The catalytic activity for the series of ZnAl_W_k__xLa and ZnAl_I_k__xLa materials is related to the La content and is primarily related to the method of doping with lanthanum. The activity of materials containing 2.8 wt% La prepared by deposition of a La compound on the surface of mixed oxides via impregnation is higher by approx. 30% as compared to materials with the same content of a dopant but obtained using the coprecipitation method. The reverse effect was found for materials with a higher content of La, i.e., 4.6 and 8.7 wt% La. For such La content, the catalytic activity of materials obtained via the coprecipitation technique was higher by approx. 40% and 50% as compared to impregnated ones. Based on the achieved results, it can be concluded that ZnO segregation on the ZnAl_2_O_4_ surface leads to a lower catalytic activity in the HT-WGS comparing to the heterojunction structure of ZnO/Zn(AlLa)_2_O_4_.

## 4. Conclusions

The way of lanthanum addition and incorporation route into the Zn–Al system plays a fundamental role in the location of lanthanum (bulk or/and surface) in final mixed oxides (ZnO/Zn(AlLa)_2_O_4_ or La_2_O_3_–ZnO/ZnAl_2_O_4_) and in creating the structure and surface morphology of the systems which determine the catalytic activity. Addition of La via coprecipitation allowed for a better distribution of La in the bulk and surface of the zinc aluminate spinel comparing to La incorporation via the impregnation technique. The right amount of La is crucial because with the lanthanum content above 2.8 wt%, distinct phase segregation of ZnAl_2_O_4_ to ZnO is observed on the surface of mixed oxides.

Deep changes in the structure of ex-ZnAl LDH materials due to calcination at 500 °C lead to the formation of heterostructural nanocomposites (ZnO/Zn(AlLa)_2_O_4_) with a structural memory. This feature reveals partial reconstruction of the original structure of a hydrotalcite analog due to impregnation by aqueous solutions of lanthanum nitrates with different concentrations.

The activity toward the HT-WGS reaction of ZnO/Zn(Al,La)_2_O_4_ and La_2_O_3_– ZnO/ZnAl_2_O_4_ mixed oxides is related to the La content. The surface ZnO segregation causes a decrease of catalytic activity in the HT-WGS. Such systems exhibit lower activity than materials containing the heterojunction structure of ZnO/Zn(AlLa)_2_O_4_ as the dominant component. However, they are base materials for designing complex multicomponent systems for a series of chemical reactions, i.e., eco-friendly catalysts for the water–gas shift reaction.

## Figures and Tables

**Table 1:** The weight loss up to 800 °C (TG) and the temperature of the highest decomposition rate (T_max_) for ZnAl_W_xLa and ZnAl_I_xLa materials.

Sample	TG (%)	T_max_ Decomposition Rate (°C)	Sample	TG (%)	T_max_ Decomposition Rate (°C)
ZnAl_W_1La	24.5	289	ZnAl_I_k__1La	12.7	164
ZnAl_W_2La	24.7	284	ZnAl_I_k__2La	13.6	153
ZnAl_W_3La	24.6	291	ZnAl_I_k__3La	13.7	199
ZnAl_W_4La	25.4	284	ZnAl_I_k__4La	14.9	206
ZnAl_W_5La	25.5	284	ZnAl_I_k__5La	16.9	240

**Table 2:** Chemical composition and textural properties of the ZnAl_W_k__xLa and ZnAl_I_k__xLa series of materials.

Sample	wt% La ^a^	(Zn/Al)_mol_^a^	S_BET_ (m^2^/g)	TPV ^b^ (cm^3^/g)	MPV_BJH_^c^ (cm^3^/g)
ZnAl_W_k__1La	0.9	0.68	158	0.34	0.34
ZnAl_W_k__2La	1.7	0.67	162	0.3	0.3
ZnAl_W_k__3La	2.4	0.7	160	0.29	0.29
ZnAl_W_k__4La	4.3	0.66	162	0.3	0.3
ZnAl_W_k__5La	8.5	0.66	154	0.32	0.32
ZnAl_I_k__1La	0.9	0.7	100	0.25	0.25
ZnAl_I_k__2La	1.9	0.68	98	0.27	0.27
ZnAl_I_k__3La	2.8	0.67	90	0.35	0.35
ZnAl_I_k__4La	4.6	0.69	89	0.27	0.27
ZnAl_I_k__5La	8.7	0.68	76	0.22	0.22

^a^ Determined by XRF; ^b^ total pore volume determined by the Barret–Joyner–Halenda method; ^c^ mesopore volume determined by the Barret–Joyner–Halenda method.

**Table 3:** The comparison of HT-WGS rate constants for the series of ZnAl_W_k__xLa and ZnAl_I_k__xLa materials.

Samples	Average HT-WGS Rate Constants k (Ndm^3^∙g_cat_^−1^∙h^−1^∙at^−0.95^)			
	350 °C	370 °C	400 °C	420 °C
ZnAl_W_k__1La	1.5	4.8	7.9	11.3
ZnAl_W_k__2La	1.9	3.1	8	11.9
ZnAl_W_k__3La	1.9	4.6	9.9	12
ZnAl_W_k__4La	2.2	4.8	10.5	12.6
ZnAl_W_k__5La	2.5	5	10.3	13.3
ZnAl_I_k__1La	2.8	5.2	9.9	12.5
ZnAl_I_k__2La	2.9	5.7	11.5	14.1
ZnAl_I_k__3La	2.3	4.9	11	13.8
ZnAl_I_k__4La	2.4	4.4	7.8	8.3
ZnAl_I_k__5La	2.3	3.5	5.6	7

## Data Availability

Data is contained within the article.

## Supplementary Materials

The following are available online at https://www.mdpi.com/article/10.3390/ma14082082/s1, Figure S1: Pore size distribution of the ZnAl_Wk_xLa and ZnAl_I_k__xLa materials; Figure S2: TEM images of the ZnAl_W_k__xLa and ZnAl_I_k__xLa materials; Figure S3: TEM images of the ZnAl_W_k__xLa and ZnAl_I_k__xLa materials; Figure S4: STEM-EDS images along with detailed maps of La (turquoise), Zn (yellow), Al (purple), and O (red) distributions of the ZnAl_I_k__1La material; Figure S5: STEM-EDS images along with detailed maps of La (turquoise), Zn (yellow), Al (purple), and O (red) distributions of the ZnAl_I_k__5La material; Figure S6: TEM images of ZnAl_W_k__xLa materials; Figure S7: TEM images of ZnAl_I_k__xLa materials; Table S1: Effect of La addition and incorporation route on the size of ZnO and ZnAl_2_O_4_ crystallites.
