# Supplementary material for: Heterostructural Mixed Oxides Prepared via ZnAlLa LDH or ex-ZnAl LDH Precursors—Effect of La Content and Its Incorporation Route
Source: Materials (Basel). 2021 Apr 20;14(8):2082. doi: 10.3390/ma14082082 (PMC8074398; doi:10.3390/ma14082082)
Supplement: Supplementary file 1 [file materials-14-02082-s001.zip › materials-1168982-supplementary.pdf]

materials

MDPI

Article

Heterostructural Mixed Oxides Prepared Via ZnAlLa LDH or ex-ZnAl LDH Precursors—Effect of La Content and Its Incorporation Route

Katarzyna Antoniak-Jurak[1*], Paweł Kowalik[1], Wiesław Próchniak[1], Robert Bicki[1], Grzegorz Słowik[2]

[1] Catalysts Department, Łukasiewicz Research Network - New Chemical Syntheses Institute, Al. Tysiąclecia Państwa Polskiego 13A, 24-110 Puławy, Poland; pawel.kowalik@ins.lukasiewicz.gov.pl (P.K.); wieslaw.prochniak@ins.lukasiewicz.gov.pl (W.P.); robert.bicki@ins.lukasiewicz.gov.pl (R.B.)
[2] Department of Chemical Technology, Institute of Chemical Sciences, Faculty of Chemistry, Maria Curie-Sklodowska University in Lublin, Maria Curie-Sklodowska Sq. 3, 20-031 Lublin, Poland ; grzegorz.slowik@poczta.umcs.lublin.pl
* Correspondence: katarzyna.antoniak@ins.lukasiewicz.gov.pl; Tel.: 4881-4731754

Table S1. Effect of La addition and incorporation route on the size of ZnO and $ZnAl_2O_4$ crystallites.

Material	d_{ZnO} (nm)	a (Å)	$ZnAl_2O_4$ (nm)
ZnAl_Wk_1La	2.5	8.01	1.3
ZnAl_Wk_2La	2.4	8.01	1.2
ZnAl_Wk_3La	2.2	8.04	1.2
ZnAl_Wk_4La	2.1	8.04	1.2
ZnAl_Wk_5La	1.7	8.08	1.2
ZnAl_I_k_1La	2.5	8.078	2.9
ZnAl_I_k_2La	2.5	8.077	2.7
ZnAl_I_k_3La	2.7	8.078	2.7
ZnAl_I_k_4La	5.7	8.083	2.9
ZnAl_I_k_5La	11.9	8.100	2.9

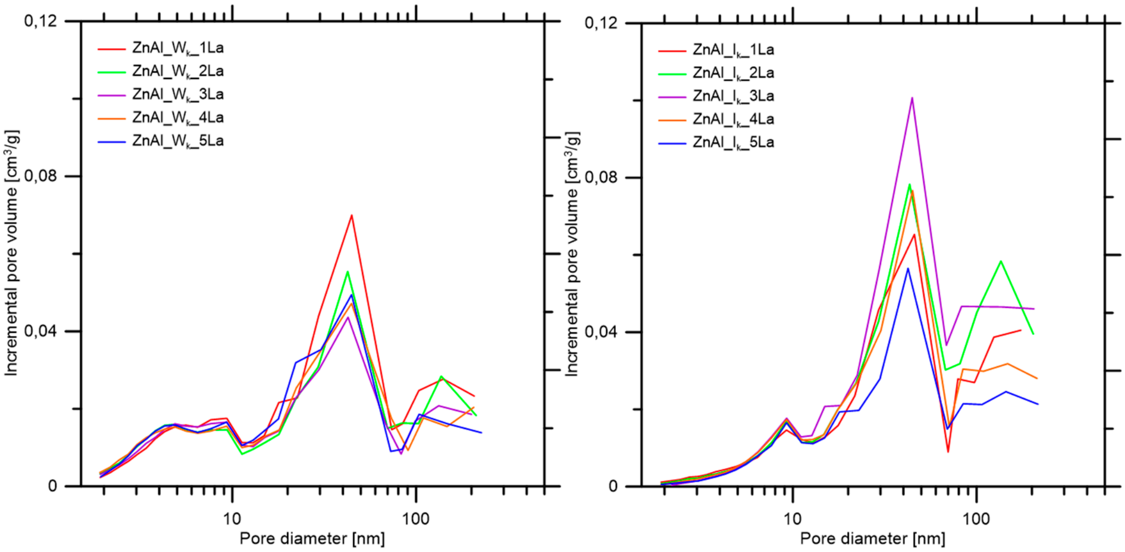

Figure S1. Pore size distribution of ZnAl_W_k_xLa and ZnAl_I_k_xLa materials.

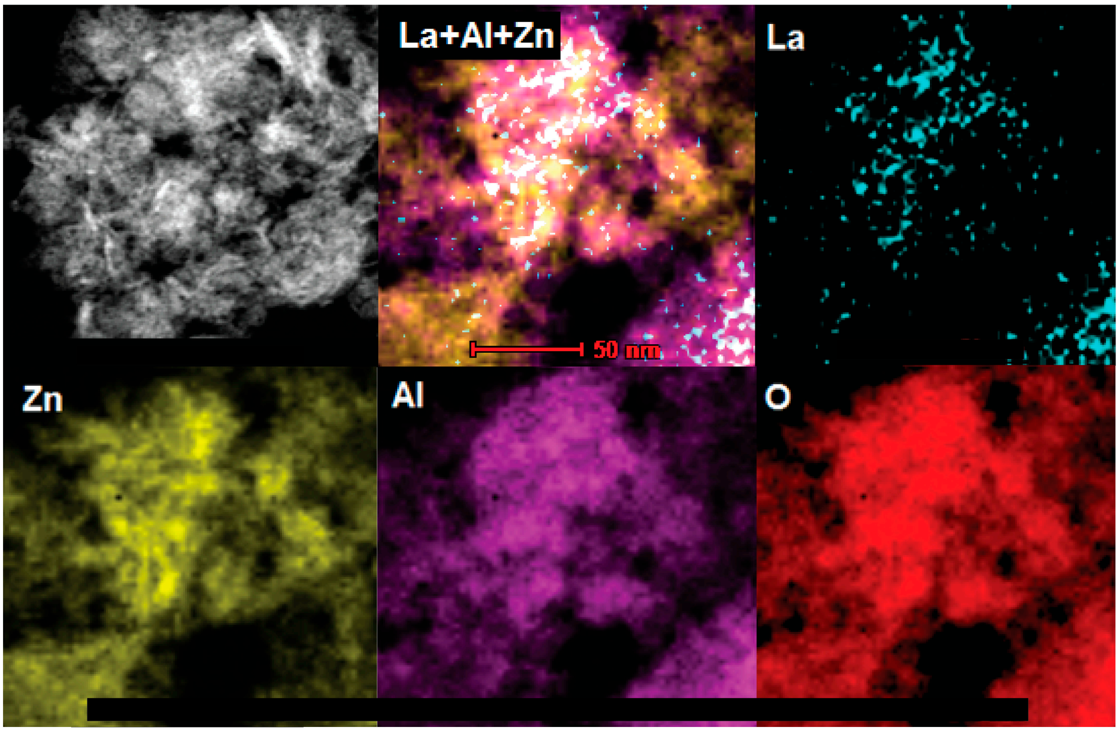

Figure S2. STEM-EDS images along with detailed maps of La (turquoise), Zn (yellow), Al (purple), and O (red) distributions of the ZnAl_W_k_1La material.

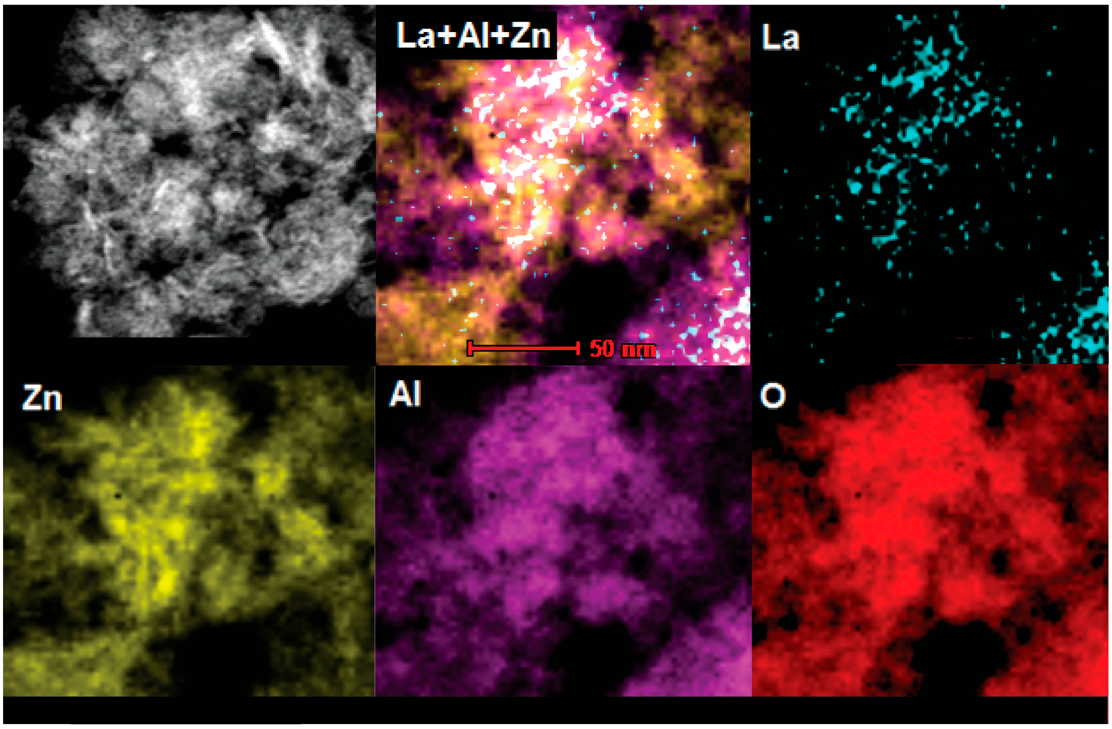

Figure S3. STEM-EDS images along with detailed maps of La (turquoise), Zn (yellow), Al (purple), and O (red) distributions of the ZnAl_W_k_5La material.

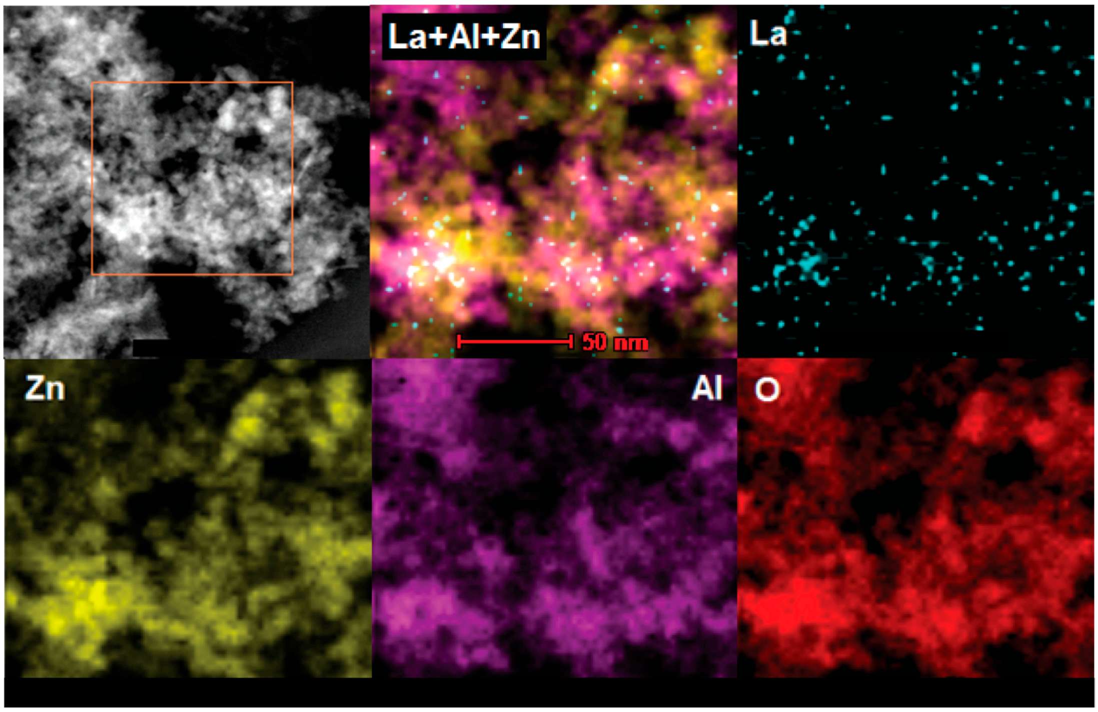

Figure S4. STEM-EDS images along with detailed maps of La (turquoise), Zn (yellow), Al (purple), and O (red) distributions of the ZnAl_I_k_1La material.

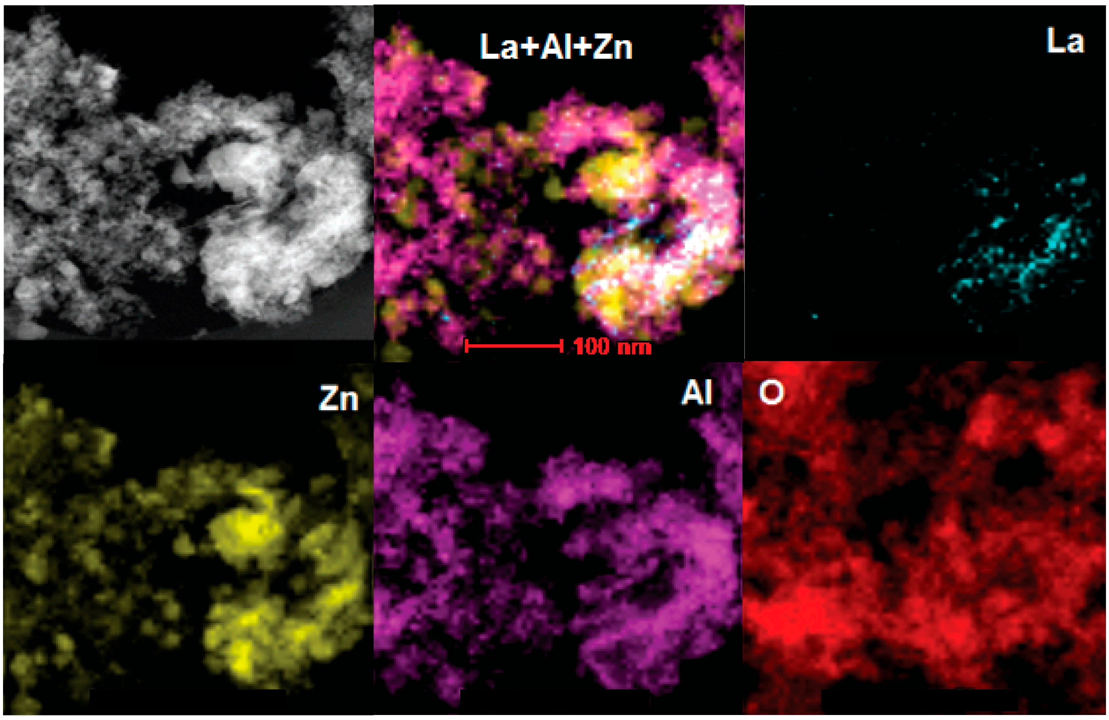

Figure S5. STEM-EDS images along with detailed maps of La (turquoise), Zn (yellow), Al (purple), and O (red) distributions of the ZnAl_I$_k$_5La material.

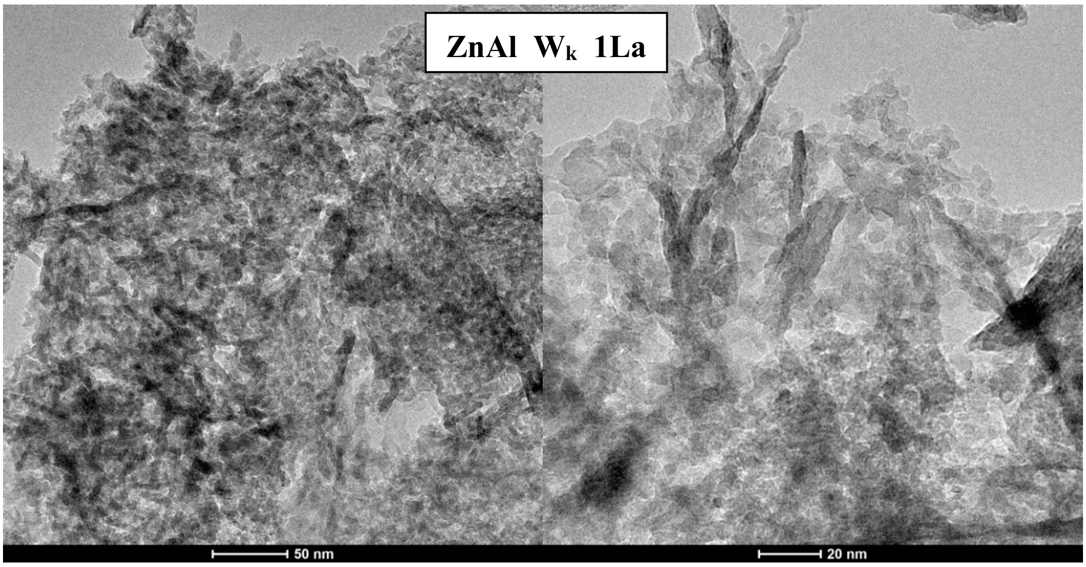

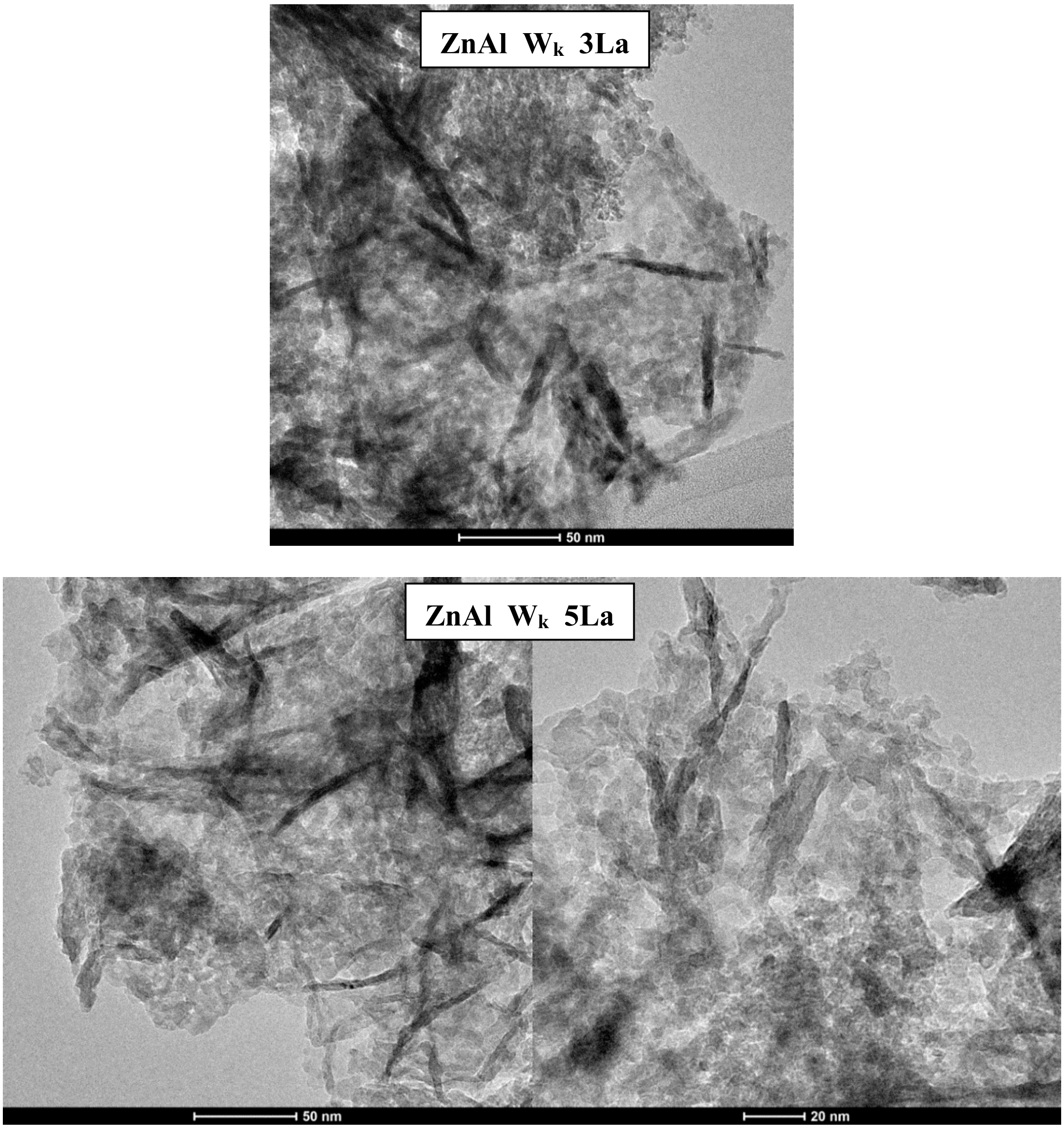

Figure S6. TEM images of ZnAl_W_k_xLa materials.

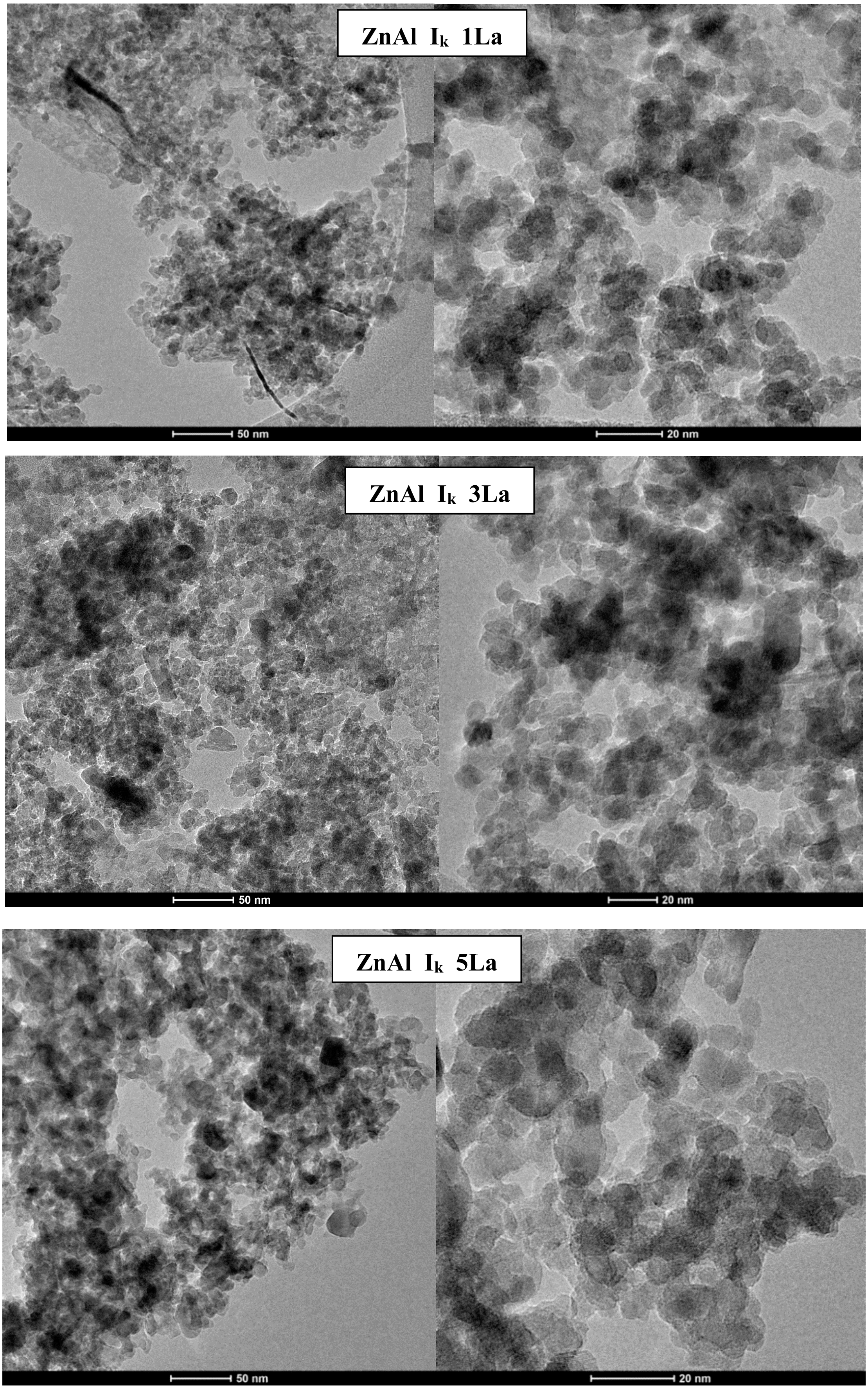

Figure S7. TEM images of ZnAl_I$_k$_xLa materials.
